# Résection laparoscopique d'une duplication gastrique chez l'adulte: traitement avec succès pour une pathologie rare

**DOI:** 10.11604/pamj.2015.22.145.7779

**Published:** 2015-10-15

**Authors:** Omar Toumi, Hiba Ben Hssine, Faouzi Noomen, Sadok Ben Jabra, Ibtissem Korbi, Ali Abdelmoula, Mayada Trimech, Wafa Ben Mansour, Boughanmi Faiez, Mohamed Ben Khlifa, Hatem Rabah, Ammar Mahmoudi, Mohamed Nasr, Khadija Zouari, Hammouda Saffar, Abdel Aziz Hamdi

**Affiliations:** 1 Service de Chirurgie Générale et Digestive, Hôpital Fattouma Bourguiba, Monastir, Tunisie; 2 Service de Gastro-Entérologie, Hôpital Fattouma Bourguiba, Monastir, Tunisie

**Keywords:** Duplication, estomac, diagnostic, endoscopie, Duplication, stomach, diagnosis, endoscopy

## Abstract

Les duplications de l'appareil digestif sont les malformations congénitales rares qui peuvent toucher tout l'appareil digestive depuis la bouche jusqu’ à l'anus. Certaines duplications sont asymptomatiques et sont diagnostiqués dans la plupart des cas pendant l'enfance. La prise en charge de la duplication gastrique est essentiellement chirurgicale. Le traitement de choix est l'exérèse complète de la duplication gastrique. Les auteurs rapportent un cas inhabituel de duplication gastrique complètement reséquée par laparoscopie. A notre connaissance, ceci est le premier cas d'une duplication gastrique traitée avec succès par laparoscopie dans la littérature Tunisienne. La Résection laparoscopique peut être ajoutée à l'arsenal thérapeutique dans le traitement chirurgical de duplications du tube digestif.

## Introduction

Les duplications digestives sont des malformations rares. Leur fréquence est estimée à 0.1 à 0.3% [1]. Le diagnostic est souvent fait pendant l'enfance, leur diagnostic chez l'adulte est exceptionnel. Cependant, quelques dizaines de cas découverts à l’âge adulte ont été rapportés [2] ce qui fait l'intérêt de notre travail. Les Duplications gastriques représentent 4% de toutes les duplications gastro-intestinales [1]. Ils sont souvent asymptomatiques et de découverte fortuite. La symptomatologie est peu spécifique ou secondaire à une complication telle que la perforation, l'hémorragie ou la compression. Le diagnostic d'une duplication gastrique est évoqué sur les examens radiologiques et confirmé par les données anatomopathologiques. Le traitement est chirurgical. Nous rapportons le cas d'une patiente ayant une duplication gastrique traitée avec succès par résection laparoscopique.

## Patient et observation

**Figure 1:**
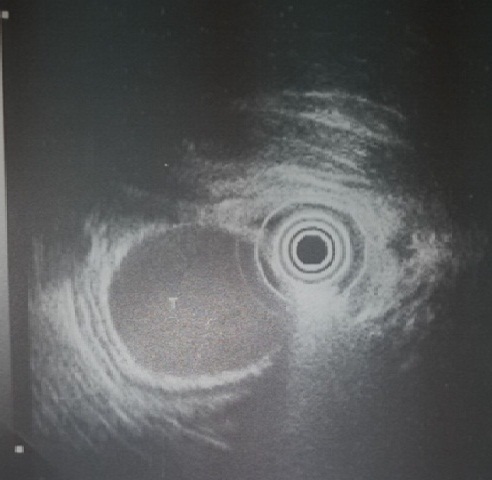
Aspect endoscopique d'une formation tumorale sous muqueuse d'allure kystique mesurant 32 mm sans atteinte des organes de voisinage ni des ganglions d'allure suspecte

**Figure 2:**
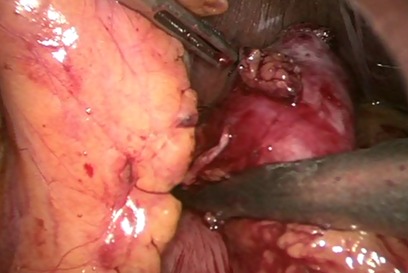
Exérèse chirurgicale réalisée à l'aide de trois applications d'une pince Endo- GIA

Patiente âgée de 23 ans sans antécédents pathologiques notables, qui présente depuis le jeune âge des épigastralgies intermittentes sans signes associés. A l'examen on notait un état général conservé, une légère sensibilité à la palpation de l’épigastre sans masse palpable. La biologie n'a pas objectivé des anomalies notamment le bilan hépatique et le bilan pancréatique. Un complément d'exploration par une fibroscopie-oeso-gastro-duodénale était effectué objectivant une tumeur sous muqueuse antrale de 03 cm de diamètre sur la grande courbure en pré-pylorique avec à la biopsie aspect d'une gastrite chronique congestive Le diagnostic d'une tumeur sous muqueuse était évoqué. Une écho-endoscopie pratiquée objectivait la présence au niveau de la grande courbure antrale d'une formation tumorale sous muqueuse d'allure kystique mesurant 32 mm sans atteinte des organes de voisinage ni des ganglions d'allure suspecte (Figure 1), l'aspect était évocateur d'une duplication gastrique. La patiente était opérée par voie coelioscopique. L'exploration confirmait la présence d'une formation sous muqueuse antrale. La masse était saisie et soulevée à l'aide d'une suspension par deux fils tracteurs permettant ainsi de bien la pédiculiser. L'exérèse chirurgicale était réalisée à l'aide de trois applications d'une pince Endo-GIA 30 (Figure 2). La pièce opératoire était extraite par l'orifice de trocart ombilical dans un sac. L'intervention a duré 60 mn avec suites simples reprise de l'alimentation à j1 post opératoire et sortie de l'hôpital à j4. La patiente était correctement suivie à notre consultation externe avec amélioration nette de la symptomatologie. L'examen anatomopathologique de la pièce avait conclu à une duplication gastrique.

## Discussion

Les duplications digestives sont des malformations congénitales rares. Les localisations les plus fréquentes sont l'iléon et l’œsophage [3]. Les duplications gastriques ne représentant que 4% des duplications digestives [1]. A notre connaissance, ceci est le premier cas de duplication gastrique traitée avec succès par laparoscopie dans la littérature Tunisienne. Leurs localisations préférentiels est la grande courbure de l'estomac [4]. Dans notre observation la duplication gastrique était plutôt localisée au niveau antrale et au dépend de la petite courbure gastrique. Pour définir la notion de duplication, ROWLING a précisé trois critères; la paroi de la duplication doit être en continuité avec l'organe dupliqué, la lumière est entourée du muscle lisse et tapissée de muqueuse digestive typique ou altérée [5]. La pathogénie reste très discutée voire obscure; plusieurs théories ont été avancées; la première est celle d'une anomalie de la fermeture latérale du disque embryonnaire; la deuxième est celle d'une origine vasculaire, sans qu'aucune ne puisse expliquer le polymorphisme de ces anomalies [1]. Sur le plan morphologique, il existe deux formes; la forme tubulaire et la forme kystique; les formes communicantes et les formes non communicantes. Cet aspect typique était trouvé dans notre observation. En effet, la majorité des duplications gastriques sont des formes kystiques, non communicantes et siègent au niveau de la grande courbure [4]. Sur le plan anatomopathologique, le kyste est tapissé par une muqueuse gastrique typique. Il peut s'y associer des ilots de tissu pancréatique ectopique [1]. L'examen anatomopathologique de la pièce de résection, a montré une muqueuse gastrique typique, sans aucun signe de dégénérescence. Cette affection touche les deux sexes de la même façon [3], et sont diagnostiqués pendant la première année de vie dans 67% des cas [3]. Les symptômes les plus fréquents sont représentés par une masse abdominale et la sténose gastrique. Moins de 25% des cas sont diagnostiqués après 12 ans. Le diagnostic d'une duplication gastrique à l’âge adulte est très rare, et peut être associée à dégénération maligne [6, 7]. Dans un tiers des cas, la DG est associée à des autres anomalies congénitales digestives tel qu'un diverticule de Meckel, un diverticule de l’œsophage, une duplication duodénale, une hétérotopie pancréatique, des anomalies extra digestives étaient également décrites comme un méningocèle ou une spina bifida [8], dans notre observation aucune de ces malformations n'a pu être détecté sur l'ensemble des explorations effectuées en préopératoire. La symptomatologie de la duplication gastrique est non spécifique, on peut noter une masse épigastrique, des douleurs abdominales vagues, une anémie ou des vomissements [9]. Le diagnostic d'une duplication gastrique peut se faire également a l'occasion d'une complication telle que la perforation, l'invagination, l'hémorragie ou la compression, la suppuration, la fistule gastro-colique, la pancréatite aigue et très rarement la cancérisation [5]. Dans notre observation, des epigastralgies non spécifiques étaient les premiers symptômes amenant à pratiquer des examens complémentaires permettant le diagnostic. Le diagnostic d'une duplication gastrique est évoqué sur les examens radiologiques et confirmé par les données anatomopathologiques. L’écho endoscopie digestive haute est plus fiable objectivant un kyste en continuité ou non avec la paroi de l'estomac avec une couche musculaire hypo-échogène parfois un péristaltisme propre à la structure kystique peut être noté [3]. L’échographie trans-pariétale, le scanner abdominal et l'imagerie par résonnance magnétique sont peu performantes et peuvent visualiser une masse para-gastrique sans pouvoir retenir le diagnostic [1]. Dans notre observation, le diagnostic de duplication gastrique n'a été retenu que par l’écho-endoscopie préopératoire. Les diagnostics différentiels qui restent à évoquer sont les pseudos kystes pancréatiques, le cystadénome, le lymphome et notamment le kyste hydatique dans un pays endémique comme le notre [5]. Vue le risque des complications, la prise en charge est chirurgicale même dans les cas asymptomatiques permettant ainsi de supprimer les symptômes et le risque très exceptionnel de cancérisation [1] D'autres modalités thérapeutiques ont été décrites allant de la réalisation, endoscopique [7] ou chirurgicale [9, 10] d'une communication large entre la duplication gastrique et la cavité gastrique jusqu’à la gastrectomie partielle voire totale [6]. Dans les très rares cas de duplications gastriques communicantes et asymptomatiques, l'abstention thérapeutique a été préconisée [9]. Il ya très peu d'articles publiés traitant la résection laparoscopique des duplications kystiques de l'estomac chez les adultes. Ce cas représente, à notre connaissance, la première exérèse laparoscopique complète d'une duplication gastrique de l'adulte rapporté en Tunisie [1].

## Conclusion

La duplication gastrique de l'adulte est une anomalie congénitale très rare. La présentation clinique est variable. Le diagnostic était établi au terme de plusieurs explorations morphologiques. Le traitement est chirurgical et consiste en une exérèse.
